# IGF1R as a Key Target in High Risk, Metastatic Medulloblastoma

**DOI:** 10.1038/srep27012

**Published:** 2016-06-03

**Authors:** Matthew N. Svalina, Ken Kikuchi, Jinu Abraham, Sangeet Lal, Monika A. Davare, Teagan P. Settelmeyer, Michael C. Young, Jennifer L. Peckham, Yoon-Jae Cho, Joel E. Michalek, Brian S. Hernandez, Noah E. Berlow, Melanie Jackson, Daniel J. Guillaume, Nathan R. Selden, Darell D. Bigner, Kellie J. Nazemi, Sarah C. Green, Christopher L. Corless, Sakir Gultekin, Atiya Mansoor, Brian P. Rubin, Randall Woltjer, Charles Keller

**Affiliations:** 1 Children’s Cancer Therapy Development Institute, Beaverton, OR USA; 2 Department of Pediatrics, Oregon Health & Science University, Portland, OR 97239 USA; 3 Division of Child Neurology, Stanford Medicine Cancer Institute, Palo Alto, CA 94304 USA; 4 Department of Epidemiology and Biostatistics, University of Texas Health Science Center, San Antonio, TX 78229 USA; 5 Division of Pediatric Neurosurgery, Department of Neurological Surgery, Oregon Health & Science University, Portland, OR 97239 USA; 6 Pediatric Brain Tumor Foundation Institute at Duke, Duke University Medical Center, Durham, NC 27705 USA; 7 Department of Pathology, Oregon Health & Science University, Portland, OR 97239 USA; 8 Departments of Anatomic Pathology and Molecular Genetics, Taussig Cancer Center and Lerner Research Institute, Cleveland Clinic Foundation, Cleveland, OH 44195 USA

## Abstract

Risk or presence of metastasis in medulloblastoma causes substantial treatment-related morbidity and overall mortality. Through the comparison of cytokines and growth factors in the cerebrospinal fluid (CSF) of metastatic medulloblastoma patients with factors also in conditioned media of metastatic *MYC* amplified medulloblastoma or leptomeningeal cells, we were led to explore the bioactivity of IGF1 in medulloblastoma by elevated CSF levels of IGF1, IGF-sequestering IGFBP3, IGFBP3-cleaving proteases (MMP and tPA), and protease modulators (TIMP1 and PAI-1). IGF1 led not only to receptor phosphorylation but also accelerated migration/adhesion in MYC amplified medulloblastoma cells in the context of appropriate matrix or meningothelial cells. Clinical correlation suggests a peri-/sub-meningothelial source of IGF-liberating proteases that could facilitate leptomeningeal metastasis. In parallel, studies of key factors responsible for cell autonomous growth in *MYC* amplified medulloblastoma prioritized IGF1R inhibitors. Together, our studies identify IGF1R as a high value target for clinical trials in high risk medulloblastoma.

Medulloblastoma is the most common malignant brain tumor in children^1^,^2^,^3^,^4^. Medulloblastoma originates in the cerebellum but tumor cells have high propensity to invade the subarachnoid space of the brain and spinal leptomeninges^5^. In standard risk patients 3 years of age or older, treatment by surgical resection, chemotherapy and craniospinal irradiation leads to greater than 80% five-year overall survival. In contrast, for infants who do not initially receive irradiation, survival is approximately 40%^6^. Nevertheless, craniospinal irradiation used to sterilize microscopic tumor cell foci in young children, even if delayed, can cause severe neurological injury and long-term survivors are challenged with life-long sequelae^7^. High risk medulloblastoma patients with gross metastatic dissemination of tumor cells to the leptomeninges experience 70% mortality rates with combined radiotherapy and intensive chemotherapy^8^ (Fig. 1A). Even with high-dose chemotherapy with autologous stem cell rescue, the five year overall survival is only 45–59% for patients with nodular subarachnoid metastases^9^.

Medulloblastoma is divided into at least four distinct molecular subgroups: WNT, sonic hedgehog (SHH), Group 3 and Group 4. These subgroups exhibit highly discriminate transcriptional, cytogenetic and mutational spectra in addition to divergent patient demographics, clinical behavior and presumed cell(s) of origin. Group 3, especially the MYC+ subgroup differs in its histopathology and gene expression profile from other forms of medulloblastoma, with a high propensity for metastasis portending a poor prognosis^10^. With respect to leptomeningeal disease, IGF1, IGF2 and IGF1R have been shown to be expressed and activated in human medulloblastoma primary tumors and leptomeningeal metastases^11^. Recently, genes associated with receptor tyrosine kinase-phosphoinositide 3-kinase signaling were found to be selectively altered in SHH medulloblastoma, including amplifications of *IGF1R*, *insulin receptor substrate 2* as well as focal deletions of *PTEN* on chromosome 10q23.31^12^. A *Ptch*+/− murine tumor model employing the Sleeping Beauty transposon mutagenesis system revealed novel mutations inducing IGF2 up-regulation and tumor formation in SHH medulloblastoma^13^. The relationship of IGF signaling, MYC and medulloblastoma pathogenesis is not surprising given the normal developmental biology of precursors of medulloblastoma. Granule neuron precursor (GNP) expansion and survival are promoted by IGF signaling^14^ and IGF signaling stabilizes the oncogenic transcription factor N-myc by inhibiting glycogen synthase kinase 3β dependent phosphorylation and consequent degradation of N-myc in cultured GNPs^15^. In support of this interrelationship, MYC over-expression combined with p53 inactivation can generate MYC-driven Group 3 medulloblastoma in the cerebellar GNPs from the external granule cell layer^16^. *MYC* amplification may be in part a driver in metastatic medulloblastoma^17^, but until recently has not itself been considered a therapeutic target. Thus, an improved biological understanding of druggable targets around this signaling axis is needed to overcome the mortality and morbidity associated with this spectrum of malignancy in medulloblastoma.

The specialized microenvironment of the brain, highly influenced by the blood brain barrier, presents a challenging yet interesting opportunity to identify the microenvironment-specific cues stimulating active or passive movement of tumor cells away from the primary site and subsequent tumor cell adhesion to the leptomeninges. Cerebrospinal fluid (CSF) flows within the subarachnoid space of the spinal meninges, winding a path around filiform arachnoid trabeculae composed of collagen cores coated with arachnoid cells. The layers of these trabeculae loosely envelope nerve roots and blood vessels^18^. Vimentin is highly expressed in these arachnoid/meningothelial cells and plays a vital role in the ultrastructural attachment to desmosomal plaques and barrier formation^19^,^20^,^21^. This arachnoid cell barrier is thought to be the target for invasion of *Neisseria meningitides* in meningitis^22^. A diagram of these structural relationships and potential interactions of medulloblastoma tumor cells is presented in Fig. 1B.

Recently, the role of secretory cytokines and growth factors from tumor cells as well as meningeal cells has gained attention^23^,^24^. In our study, we investigated cell autonomous survival factors in medulloblastoma as well as cytokines and growth factors that may be responsible for chemotaxis towards the leptomeningeal membrane.

## Results

### Identification of CSF Cytokines, Growth Factors, and Growth Factor Receptor Expression in Medulloblastoma Clinical Samples

**Figure 2:**
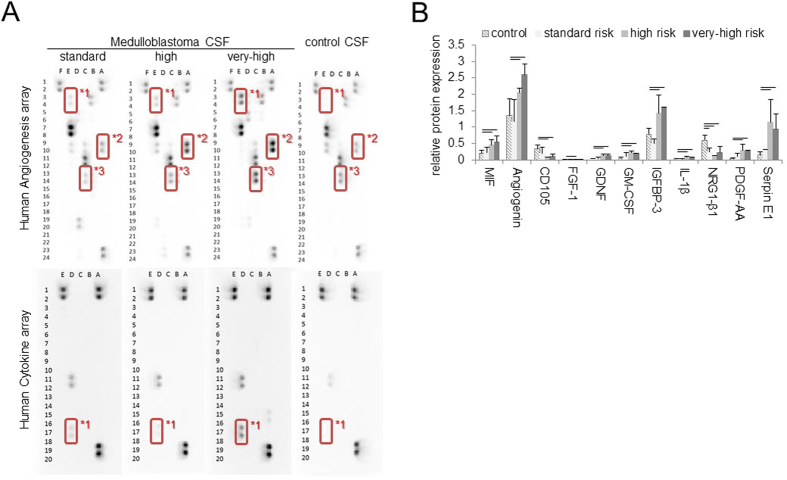
Cytokines and Growth Factors from CSF samples. (**A**) Representative proteome profile array results for medulloblastoma patient derived CSF versus acute lymphoblastoma leukemia CSF. (**B**) Summary of differentially expressed proteins for the human cytokine profile arrays for CSF samples. See also Supplemental Figure S1. *1 SerpinE1/PAI-1. *2 Angiogenin. *3 IGFBP3. Horizontal bars represent comparisons for which P < 0.05.

To determine the potential clinically-relevant cytokines and growth factors in metastatic medulloblastoma, we performed filter-based hybridization arrays on CSF from pediatric patients with medulloblastoma. Risk category was assigned based upon age, extent of resection (greater than or less than 1.5 cm^2^ residual primary tumor, and presence of metastases by lumbar CSF cytology and spine MRI, and the histologic presence of moderate or severe diffuse anaplasia. For controls, CSF was collected from pediatric acute lymphoblastic leukemia (ALL) patients, none of whom had CSF leukemic involvement during their disease course (Table 1). Representative results are shown in Fig. 2A and findings are summarized in Fig. 2B. Relative to controls, a risk-related increase in protein expression was seen for SerpinE1/PAI-1 (denoted *1), Angiogenin (denoted *2) and IGFBP-3 (denoted *3). TIMP1 was also highly expressed in all samples (Supplemental Fig. S1). A full summary of all other cytokines and growth factors measured is given in Supplemental Fig. S1. In light of elevated IGFBP levels, we also assessed total levels of IGF1 from CSF samples. Previous reports of normal pediatric CSF levels for children younger than 6 months old (mo) was 10.8+/−0.6 ng/ml, whereas for children 6 mo and older the IGF1 levels were reported as 6.9+/−0.6 ng/ml^25^. In our CSF samples for patients all >6mo, high risk medulloblastoma patient IGF1 levels were significantly higher than ALL patient IGF1 levels (9.56+/−4.49 versus 3.47+/−2.09, p = 0.04) (Table 1). We next examined mRNA expression of *IGF1, IGFBP3, MMP9, SERPINE1*, *TIMP1* compared to normal cerebellum using publically available datasets. For these genes, probes were significantly differentially expressed across samples as determined by Student’s t-test in medulloblastoma samples for *IGF1R* (5/ 5), *SERPINE1* (7/7). Significant differential expression of *IGF1* and *IGFBP3* was seen only in 2 of 5 and 1 of 2 probes respectively. No differential expression was observed for *MMP9* or *TIMP1*. Expression of these genes did not show a pattern consistent with the different medulloblastoma subtypes, possibly indicating these genes as commonly differentially expressed in medulloblastoma (Fig. S3A). ELISA performed on these paired samples revealed IGF1 levels of 5.2 ng/mL in the primary tumor, 5.28 ng/mL in the secondary tumor and 5.36 ng/mL in the meninges which are elevated levels relative to normal CSF (Table 1) and support the assertion that elevated IGF1 levels can occur at the leptomeningeal surface.

### Validation of a Meningothelial Model System

To perform functional studies, we required models of both leptomeningeal cell cultures and metastatic medulloblastoma cultures. Meningioma tumor cells, thought to have arisen from arachnoid/meningothelial cells, are a model of normal leptomeningeal cells for *in vitro* studies of meningitis^22^,^26^. Meningioma cells not only model bacterial attachment properties but also the strong cytokine response of the meningothelium that is both attachment-dependent and attachment-independent^26^. Meningiomas have a range of histologies, from the more benign fibroblastic appearance characterized by fibroblastic markers (Vimentin) to the more carcinomatous and invasive variants that express epithelial markers^19^. The former are especially difficult to culture because these benign variants senesce unless immortalized^20^.

**Figure 3:**
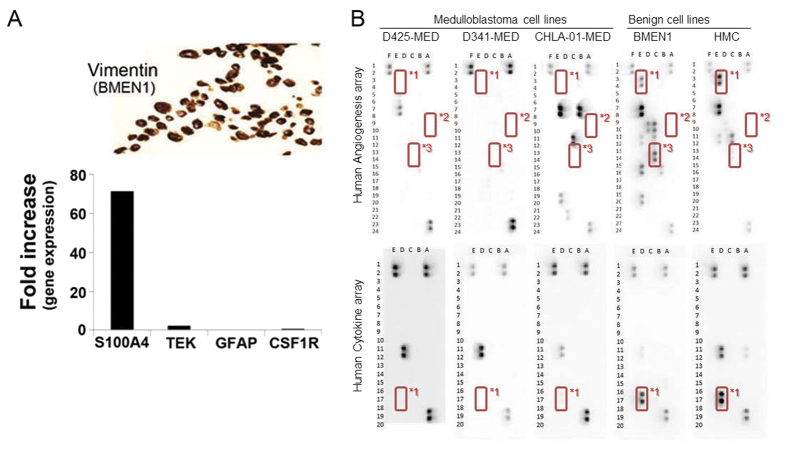
Cytokines and Growth Factors from MYC+ medulloblastoma conditioned media compared to a meningothelial cell model. (**A**) The benign meningioma cells (BMEN1) used in this study to model normal meningothelial cells were immunostained for the vimentin protein, a meningioma cell marker (Fig. 3A, upper), as well as compared with the normal human meningeal cells (HMC) for the expression of a meningioma/fibroblast marker (*S100A4*), endothelial cell (*Tie2*), glial cell marker (*GFAP*) and the macrophage marker (*CSF1R*) by RT-PCR. Fold increase in BMEN1 cells versus HMC is represented as the bar graph (Fig. 3A, lower). (**B**) Representative proteome profile array results for medulloblastoma cell lines and BMEN1 conditioned media. (top) Summary of differentially expressed proteins for the human angiogenesis profile arrays. (bottom) Summary of differentially expressed proteins for the human cytokine profile arrays. *1 SerpinE1/PAI-1. *2 Angiogenin. *3 IGFBP-3.

In our experimental system we first compared an immortalized benign meningothelial meningioma cell line (BMEN1^27^) to a commercially-available bulk preparation of cells stripped from the human leptomeninges (human meningeal cells, HMCs). S100A4 (also called FSP-1) can be expressed in astrocytes^28^ but is most widely known to be a marker of mesenchymal cells as well as meningiomas^29^. This mesenchymal/fibroblastic marker was expressed 70-fold higher at the transcript level in BMEN1 cells than HMC bulk-prepared cells (Fig. 3A, lower), suggesting that BMEN1 is a specific model of the arachnoid/meningothelial cells. Markers of the endothelium, astrocytes and macrophages were comparable between BMEN1 and HMC cells (Fig. 3A, lower). Vimentin was also strongly expressed by immunocytochemistry in BMEN1 cells (Fig. 3A, upper).

### Identification of CSF Cytokines and Growth Factors in MYC+ Medulloblastoma Cell Lines

We next validated MYCC or MYCN amplification by FISH for selected medulloblastoma cell lines from metastatic medulloblastoma patients (Table 2; Supplemental Fig. S1). We then examined the pattern of abundance of cytokines and growth factors in conditioned media from cell lines (Fig. 3B). Expression of SerpinE1/PAI-1 (*1), Angiogenin (*2) and IGFBP-3 (*3) were notably low in medulloblastoma conditioned media, but SerpinE1/PAI-1 and IGFBP-3 were in fact present in BMEN1 cells. For HMC cells, SerpinE1/PAI-1 was the most highly expressed. TIMP1 was also expressed at relatively high levels in BMEN1 and HMC cells, but also CHLA-01-MED cells.

### Validation of a Bioactive, Ligand-dependent IGF1R Axis in Medulloblastoma

**Figure 4:**
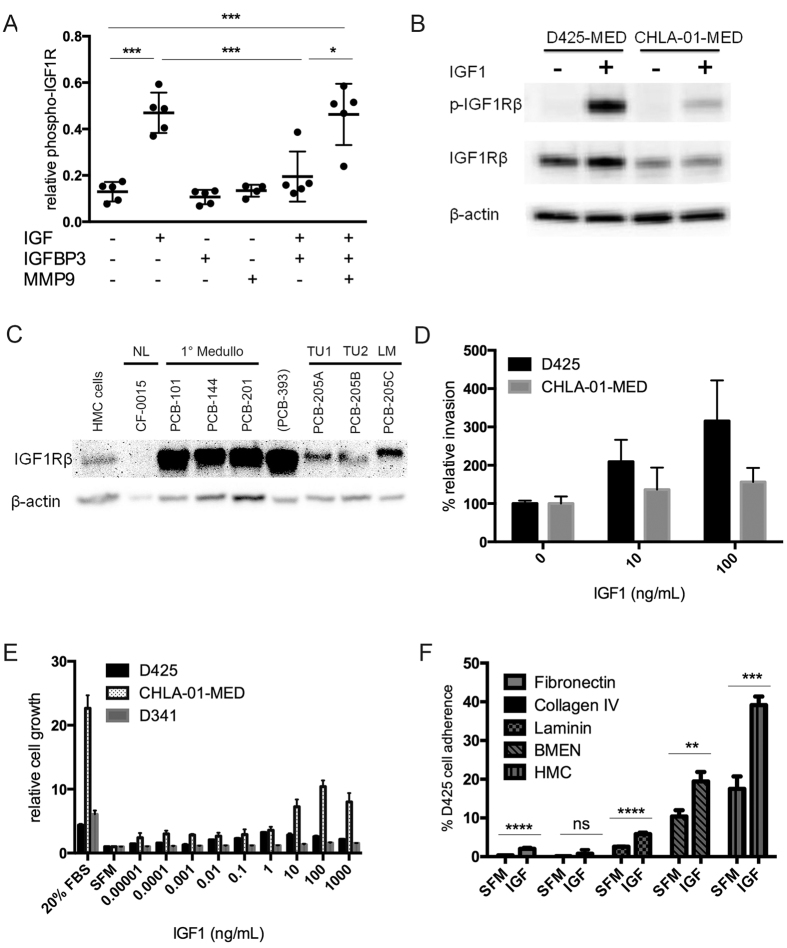
IGF1 induces migration, mitogenesis, and adhesion of medulloblastoma cell lines. (**A**) Cleavage assay in D425 cells examining bioactivity of IGF1 cleaved from IGF1-IGFBP3 complexes by MMP9. (**B**) Immunoblotting of IGF1R and phospho-IGF1R in D425 and CHLA-01-MED cells after 10 min IGF1 (100 ng/ml) stimulation. (**C**) Migration assay of D425 and CHLA-01-MED cells using a polyester Boyden chamber. Black and gray lines show significant difference (P < 0.05). (**C**) IGF1R overexpression in medulloblastoma primary tissue relative to normal cerebellum (CF-0015) and meningothelial cultured cells (HMC). Medulloblastoma samples were PCB-101 (8yo male, MYC negative by FISH, from surgical biopsy), PCB-201 (4yo male, MYC negative, anaplasia, from surgical biopsy), and three samples from a metastatic patient, PCB-205 (4yo male, MYC negative, from autopsy). Paired samples from PCB-205 consisted of a primary tumor (205A), a secondary metastatic tumor (205B), and microscopically negative meninges (205C). See Results for further description. Sample PCB-393 was from a cerebellar glioblastoma. (**E**) MTS assay of D425, D341-MED and CHLA-01-MED cells after stimulated by IGF1. Black, gray, and dashed lines shows significant difference (P < 0.05). (**F**) Adherence assay of D425 in pre-coated plates containing the extracellular matrix components, fibrin, collagen IV, and laminin or cell culture models of the leptomeninges. A p ≤ 0.05 is indicated by * and *** indicates p < 0.001.

Prior reports have suggested that IGFBP3 increases IGF1 ligand bioavailability in the context of cleavage of IGFBP3 by metalloproteases^30^,^31^. To confirm the biochemical activity of MMP9 and the bioactivity of free IGF1 cleaved from IGF1/IGFBP3 complexes in a medulloblastoma cell culture model system, we performed an MMP9 cleavage assay wherein IGF1/IGFBP3 complexes were exposed to MMP9 in a molar substrate:enzyme ratio of 1:1 and measured phospho-IGF1 by ELISA in D425 cells. In the absence of MMP9, no significant difference was observed between a serum-free media control and the IGF1/IGFBP3 treatment group. This finding suggested a physiologically-relevant attenuation of IGF1 bioactivity in the presence of IGFBP3. However, in the presence of MMP9, a statistically significant increase in IGF1R phosphorylation was observed for the IGF1/IGFBP3/MMP9 treatment group compared to a serum-free media control. Indeed, the magnitude of this phosphorylation induction for the IGF1/IGFBP3/MMP9 treatment group was comparable to the IGF1 alone group. Thus, in the context of MMP9 mediated cleavage of IGF1 from IGFBP3, IGF1 bioactivity is not significantly attenuated (Fig. 4A). We then extended the observation that c-MYC amplified medulloblastoma was biochemically responsive to IGF-1 stimulation in D425 cells to CHLA-01-MED cells (Fig. 4B). Immunoblot analysis of primary patient-derived medulloblastoma tumor samples demonstrated IGF1 receptor overexpression in 100% of samples surveyed (n = 5) as compared to normal cerebellum and a meningothelial cell culture. Of note, one sample also showed IGF1R expression present in a matched set of primary tumor, secondary tumor and microscopically-uninvolved meninges. Histology of the meninges confirmed the presence of focal hemosiderin, histiocytes and lymphocytes (Figs 4C and S3C,D) suggesting that meninges in disease conditions themselves may utilize IGF1R signaling and may have need of regional IGF1 bioavailability.

To examine whether IGF1 bioactivity induced migration and adherence, independent of mitogenesis, we first examined the invasive potential of D425 and CHLA-01-MED cells exposed to IGF1. Doses of 10 and 100 ng/ml led to 1.4–2 to 1.5–4 fold increases in migration in Boyden chamber assays relative to serum-free media (Fig. 4D). As expected, these doses were also mitogenic (Fig. 4E). We next examined the adherence potential of D425 cells to the leptomeningeal extracellular matrix components laminin, fibronectin, and collagen IV as well as the cell-cell adhesion potential of D425 cells to the human leptomeningeal cell culture models BMEN and HMC cell lines. A dose of 10 ng/mL led to increased adhesion to the extracellular matrix components fibronectin and laminin with fold increases of 5.3–7.7 and 1.9–2.7, respectively. Similarly, following exposure to IGF1 increased adhesion of D425 cells BMEN and HMC cells with observed fold increases of 1.9–2.7 and 1.2–1.4, respectively (Fig. 4F).

### Meningothelial Cells are a Source of IGFBP-cleaving Proteases

**Figure 5:**
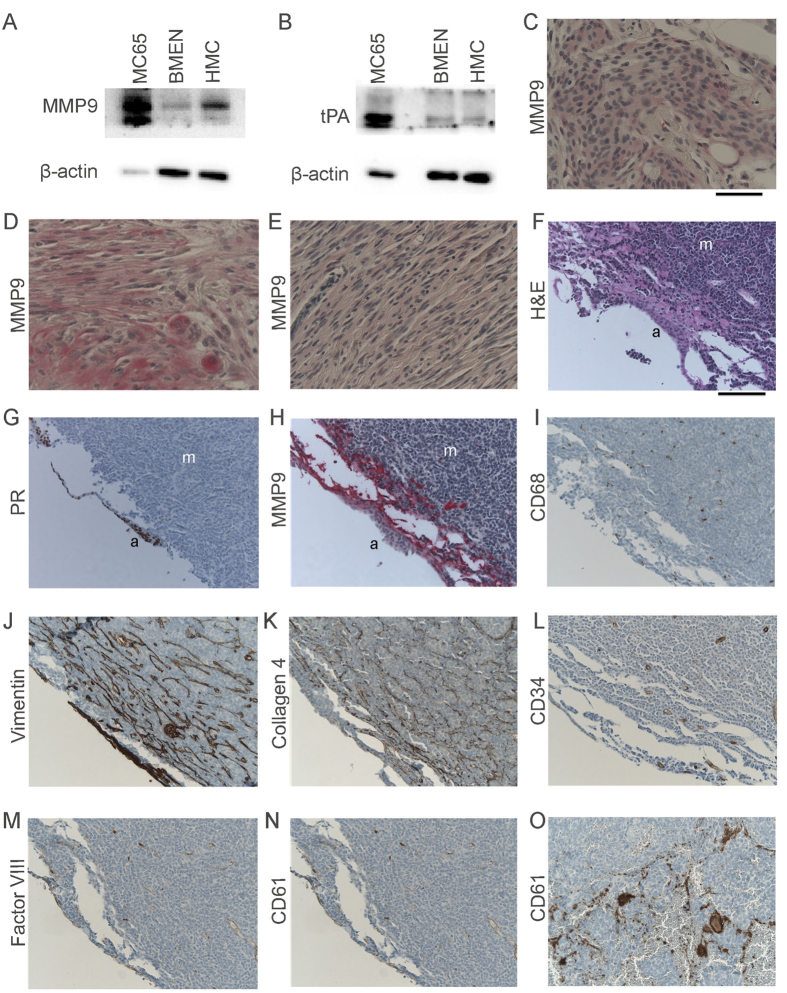
Protease expression in cells of leptomeningeal origin. (**A,B**) Protein expression of MMP9 and tPA in BMEN1 and HMC cells with neuroblastoma cells as a positive control^47^. (**C–E**) Expression of MMP9 in meningiomas. MMP9 expression in human meningeal tumors was determined by immunohistochemistry performed on low-grade meningiomas with a variety of morphologies. MMP9 was variably expressed in all meningiomas tested (n = 10), with greater expression in epithelioid than spindly tumors. Typical expression levels are seen in (**C**) levels in more epithelioid (**D**) and spindly (**E**) are also displayed. Vector Red was used as the chromogen in each panel. scale bar, 50 μM. (**F–O**) Expression of MMP9 and other markers in medulloblastoma-associated meninges. Meningeal surfaces adjacent to medulloblastomas were often difficult to identify by routine histologic stains (**F**), but could reliably be located with the use of anti-progesterone receptor, PR (**G**). Modest MMP9 expression was present in meningothelial cells at similar levels observed in meningiomas. More striking expression observed in the basal arachnoid and in the subjacent tumor (**H**). MMP9 expression here was comparable to that observed in areas of degenerating medulloblastoma with macrophage infiltration (not shown). However, CD68 immunostain demonstrated no macrophages, but only occasional tumoral microglia in these regions (**I**). Vimentin (**J**) and Collagen 4 (**K**) immunohistochemistry revealed increased tumor stroma in a submeningeal distribution within the medulloblastoma, but CD34 (**L**) and Factor VIII (**M**) immunohistochemistry did not demonstrate increased tumor vascularity in this region. Anti-CD61 immunostain for thrombocytes did not reveal hemorrhage or its reorganization as a source for MMP9 in this region (**N**) despite positive staining in thrombosed intratumoral vessels deeper elsewhere (**O**). Vector Red was used as chromogen in (**C–E,H**); all other immunohistochemical stains were developed using diaminobenzidine. Original magnification of all images was 200x. a, arachnoid cells. m, medulloblastoma tumor cells. scale bar, 100 μM.

To consider the possibility that meningothelial cells may be a source of IGFBP-cleaving proteases, we performed western blotting of MMP9 and tPA on cultured BMEN and HMC cells, using cultured human neuroblastoma (MC65) cells as a positive control for MMP9 expression. Both BMEN and HMC cells were found to express both proteases, depicted in Fig. 5A,B. As we had previously validated meningiomas as a cell culture model system of the leptomeninges, we sought to determine whether MMP9 was expressed in human meningothelial-derived tumors by immunohistochemistry as a surrogate for reactive leptomeninges as might be present in medulloblastoma. Low-grade meningiomas display a variety of morphologies, ranging from epithelioid variants that more closely resemble non-neoplastic meningothelial cells to more spindle cell-like tumors. A survey of meningiomas from 10 patients demonstrated MMP9 expression in all of these, with higher levels of expression in more epitheloid tumors and in the epithelioid areas of tumors with mixed morphologies (Fig. 5C–E).

We next determined expression of MMP9 associated with meninges from 15 patients who underwent resection of medulloblastoma (Fig. 5F–O). Immunohistochemistry for progesterone receptor, which is strongly expressed in meningothelial cells, was used to identify interfaces of tumor and meninges unambiguously. Modest MMP9 expression, similar to that observed in meningiomas, was observed in meningothelial cells; however, markedly increased expression was identified on the basal aspect of the meninges and in the immediately subjacent tumor. Expression in these regions was comparable to strong expression seen in areas of degeneration of tumor containing macrophages, which are known to express MMP9 strongly. However, CD68 immunostain did not demonstrate macrophages in MMP9-expressing regions adjacent to meninges. Rather, increased expression of the stromal antigens Vimentin and Collagen IV were present in these areas. These appear to originate from leptomeningeal-derived tumor cells, rather than vascular stroma, as no increased vascularity or vascular thrombosis was observed in these regions by immunohistochemistry for CD34 or factor VIII (endothelial), or CD61 (thrombocyte) antigens.

### Selection of High Priority Single Agent Targeted Therapeutics Suppressing Cell-Autonomous Growth in High Risk Medulloblastoma

**Figure 6:**
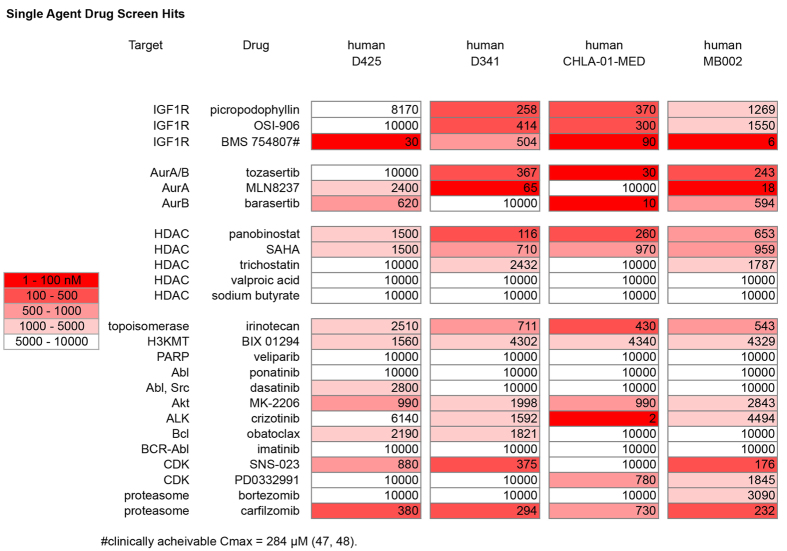
Single Agent Drug Screen Hits. Heat map demonstrating medulloblastoma cell line sensitivity to growth inhibition to select agents in a panel of sixty investigator-selected small molecule compounds. The values shown are the absolute IC50s. Target and drug name are given to the left and cell lines are indicated above.

To establish the relative prioritization of targeted small molecule inhibitors for the inhibition of cell growth in metastatic medulloblastoma, we performed a 60-compound screen of agents heavily weighted towards contemporary pediatric phase I and phase II pipelines. Compounds are listed in Supplemental Tables S1, and results of the screen for D425, D341 and CHLA-01-MED cell lines are given in Fig. 6. We also performed this same screen on an autopsy-derived primary cell culture from a 4yo with disseminated Group 3 MYC+ medulloblastoma (MB002), also presented in Fig. 6. Consistent bioactive agents included aurora kinase inhibitors, histone deacetylase inhibitors, and IGF1R inhibitors.

## Discussion

Presumed micro-metastatic seeding of tumor cells on the leptomeninges is a primary reason that craniospinal irradiation is included as a mainstay in the treatment of medulloblastoma. Unfortunately, this irradiation can leave life-long sequelae. When disease progression occurs, the pattern of relapse includes leptomeningeal spread or brain metastasis in approximately 80% of cases, and metastasis can occur with or without local recurrence^8^. Therefore, understanding the molecular mechanism regulating migration and adhesion of tumor cells on the leptomeninges and identifying microenvironment-specific novel targets is crucial for therapeutic interventions aimed at overcoming the mortality and morbidity associated with medulloblastoma and its treatment. The biology of tumor cells that leave the CSF appears to be also divergent, in that patients with free-floating viable tumor cells in the CSF (Chang operative stage M1) have improved outcomes with radiotherapy before chemotherapy, but for patients with nodular intracranial subarachnoid or spinal subarachnoid seeding (Chang operative stages M2 and M3, respectively), treatment with irradiation before chemotherapy leads to worsened survival^8^. In an effort to understand these phenomena, careful clinical phenotyping has led to the recognition of three biologically distinct forms of metastases, namely free-floating tumor cells in the CSF, nodular metastases and laminar (with or without nodular) metastases. The 5 year overall survival for these patients after high dose chemotherapy/autologous stem cell rescue has been reported as 47%, 59% and 35%, respectively^9^. The lowest survival, therefore, is for patients with adherent thin coating of tumor cells to the subarachnoid space. Possible microanatomical reasons for this mortality may be interference with CSF function and microcirculation, altering transport of molecules between blood and CSF as well as CSF and brain through the pial surface, although increased mortality in these patients may also reflect underlying genetic characteristics of the tumor that result both in laminar spread and an independent tendency towards therapeutic refractoriness.

**Figure 7:**
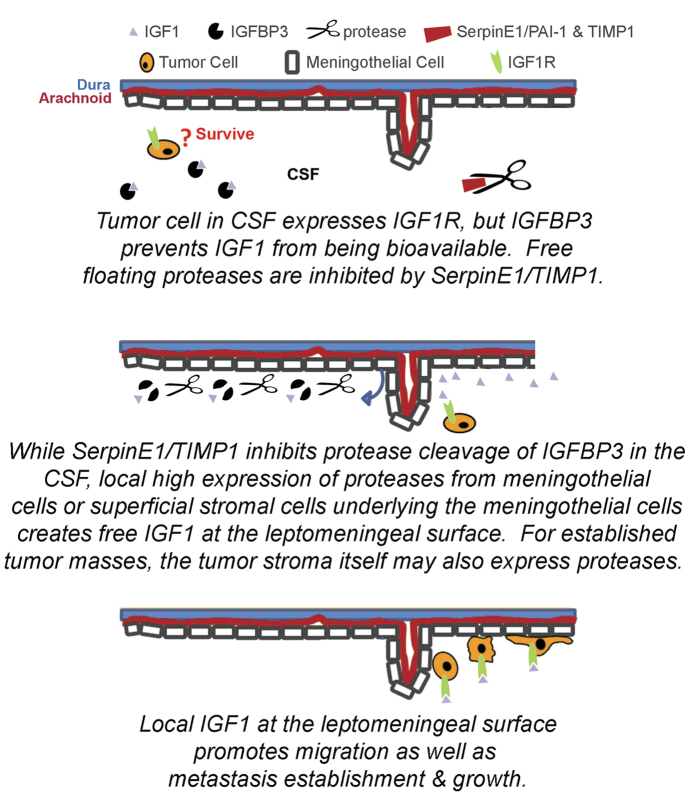
Model for IGF1-mediated Leptomeningeal Metastasis. In our proposed model, bioavailability of IGF1 is highest at the leptomeningeal surface, where IGF1 can be locally liberated by IGFBP3-cleaving proteases. Metalloproteases including MMP-9 are known to be expressed not only in the brain parenchyma under physiological or diseased states but also by the uninjured or injured leptomeninges^48^,^49^,^50^ and tPA is known to be expressed from the both meninges as well as the vertebral artery^51^,^52^. Both TIMP1-MMP-IGFBP3-IGF and SerpinE1/PAI-1-tPA-IGFBP3-IGF axes have been previously described in other model systems^30^,^31^,^34^.

When considering intracranial or spinal metastasis at the cellular level, one presumes that since medulloblastoma develops in the cerebellum, metastasis requires tumor cells to move (passively and/or by active migration) through the ventricular and CSF spaces and to adhere to meningothelial cells; however, the signaling cues regulating these processes are not known. At least from the tumor cell perspective, an Igf2-Akt axis has been implicated in experimental mouse models^32^. To explore the mechanisms of leptomeningeal metastasis, we first explored expression of cytokines and growth factors in CSF from patients with metastatic and non-metastatic medulloblastoma. Notable were expression of IGF1, IGF1-chaperone IGFBP3, the protease inhibitor SerpinE1/PAI-1 and the metalloprotease inhibitor TIMP1. In metastatic tumors, the fibrin matrix that might be induced by SerpinE1/PAI-1 forms a rudimentary scaffold that is an absolute requirement for supporting the growth of the tumor itself and the incoming new vessels^33^. In other reports, the co-expression of IGFBP3 and a serine protease/tPA inhibitor (SerpinE1/PAI-1) or a metalloprotease inhibitor (TIMP1) has been suggested to modulate IGF ligand bioavailability^30^,^31^,^34^. Our model, supported by experimental observations, for how IGFBP3 and SerpinE1/PAI-1 or TIMP1 might promote IGF1 bioavailability at the leptomeningeal surface is given in Fig. 7. We propose that tumor cells in CSF express IGF1R, but IGFBP3 prevents IGF1 from being bioavailable. Furthermore, IGF1 may be a growth factor required for survival of tumor cells in the CSF. While SerpinE1/TIMP1 inhibits protease cleavage of IGFBP3 and IGF1 liberation in the CSF, free floating proteases in the CSF are inhibited by SerpinE1/TIMP1; however, local high expression of proteases from meningothelial cells or superficial stromal cells underlying the meningothelial cells creates free IGF1 at the leptomeningeal surface. For established tumor masses, the tumor stroma itself may also express proteases. Meningothelial cells may not be a significant barrier to medulloblastoma cells. With invasion of medulloblastoma cells, it is likely that the superficial stromal cells become exposed to a host of additional factors from the highly vascular meninges and from the CSF. Invasion of these structures may induce MMP9 expression in nearby tumor stromal cells. If medulloblastoma cells respond to IGF1, this would provide a “feed-forward” mechanism for tumor growth once the tumor reaches the leptomeninges. Similarly, local invasion of inflammatory cells may contribute further to the regional IGF concentration within the milieu of the leptomeninges.

Supporting this model were our functional studies of MYC+ medulloblastoma cell lines suggesting that IGF1 was associated not only to IGF1R phosphorylation and increased cell viability, but also migration and adhesion to the extracellular matrix components and cell types associated with the leptomeninges. Cell viability screening of targeted small molecule inhibitors further prioritized IGF1R inhibitors for the metastatic, *MYC* amplified medulloblastoma tumor cell cultures. These results are consistent with prior studies by our own group demonstrating IGF1R to be a therapeutic target in primary medulloblastoma tumorspheres from a transgenic mouse model of medulloblastoma^35^ as well as reports of IGF1R expression in human medulloblastoma^36^. Furthermore, prioritization of insulin signaling as a therapeutic target in medulloblastoma has been proposed by Wu *et al.* Their recent studies suggest a bicompartmental model of leptomeningeal metastasis whereby restricted metastatic clones arise via clonal selection of the primary tumor and that PI3 kinase signaling contributes to medulloblastoma progression and metastasis (29). The practice of craniospinal radiation underlines the assumption of micrometastatic tumor cell seeds being present at diagnosis; furthermore, the pattern of failure for Group 3 and 4 medulloblastoma is frequently metastasis without primary site recurrence^37^. In either case, the child who has detectable metastasis may be the most important patient to treat with an anti-metastasis therapy since the patient’s tumor cells are proven capable of creating metastases: preventing a cascade of new metastases while also inducing regression of established metastatic tumors may simultaneously be required to prevent fulminant disease and demise. Here we provide complementary evidence implicating IGF1 signaling as a potential driver of metastatic dissemination throughout the cerebrospinal axis.

Future studies will be aimed at dissecting the sensitivity of the metastatic compartment and metastatic lesions to small molecule inhibitors of IGF1R. It is likely that an *in vivo* imaging model and a fluid dynamic cell model system will need to be established to address metastatic compartment sensitivity to IGF1R inhibition and the complex, dynamic interactions that regulate chemokine bioavailability for medulloblastoma metastasis to the leptomeninges. The model proposed in Fig. 7 is one such set of interactions that require an optimized fluid dynamics approach and the multidisciplinary integration of engineering and biologist research teams. As precision medicine becomes an ever-present theme, the efforts at institutional and cooperative group levels to establish patient-derived primary cell cultures and xenografts for MYC+ medulloblastoma patients may inform these efforts even further.

## Experimental Procedures

### CSF and Tumor Samples

Human CSF samples from either clinical external ventriculostomy drains present for management of hydrocephalus in the peri-operative period and/or from lumbar thecal CSF sampling for tumor staging were obtained by informed consent under an Institutional Review Board (IRB)-approved protocol at OHSU. The autopsy-derived primary cell culture from a 4yo with disseminated Group 3 MYC+ medulloblastoma (MB002) was generated under IRB approval of Stanford University and carried out in accordance with IRB guidelines for human subjects research. Patient-derived tumor samples were collected from patients undergoing planned surgical resection or research-autopsy who had enrolled in the biobanking study the Childhood Cancer Registry for Familial and Sporadic Tumors (CCURE-FAST). All patients provided informed consent, patient data and clinical and pathologic findings were maintained in a de-identified database. All aspects of the study were reviewed and approved by the Oregon Health & Science University Institutional Review Board and carried out in accordance with OHSU IRB guidelines for human subjects research.

### Cytokine & Angiogenesis Arrays

The expression profiling of angiogenesis-related proteins in the supernatant conditioned media from BMEN-1 and medulloblastoma cells lines were performed according to the manufacturer’s protocol (proteome profiler human cytokine and human angiogenesis array kits, R&D Systems, Minneapolis, MN). The membranes were incubated in the manufacturer-provided blocking buffer and 250 μl CSF or 1 ml conditioned media were mixed with the cocktail of biotinylated detection antibodies for 1.5 hr before adding to the membranes and incubation continued overnight at 4 °C. Membranes were washed and incubated with streptavidin-conjugated horseradish peroxidase and chemiluminescence was detected using IVIS Lumina II imaging system (Caliper Life Sciences, Hopkinton, MA). Statistical comparisons were made by the Student’s t-test.

### ELISAs

Total IGF1 and IGFBP3 in CSF was quantified by an ELISA according to the manufacturer’s protocol (Quantikine ELISA, R&D Systems). In order to release IGF1 from IGF binding proteins, patient-derived CSF samples were pretreated with an acidic dissociation solution. Standards and pre-treated samples were then added to the wells of a microplate pre-coated with an IGF1or IGFBP3 monoclonal antibody and incubated for 2 hours at 4 °C. Wells were aspirated and washed and 200 μL of IGF1or IGFBP3 conjugate, containing an IGF1or IGFBP3 polyclonal antibody conjugated to horseradish peroxidase, was added to each well and incubated for 1 hour at 4 °C. After an additional wash, 200 μL of substrate solution containing a tetramethylbenzidine chromogen was added to each well and incubated for 30 minutes at room temperature. Following color development, a stop solution of 2N sulfuric acid was added to each well and optical density was determined using a microplate reader set to 450 nm with wavelength correction set to 570 nm.

### Cell Culture and Conditioned Media Preparation

D425 has been previously reported^38^ and was obtained from co-author DDB. D341^39^ and CHLA-01-MED were obtained from ATCC (CRL-3021 and HTB-187, respectively; ATCC, Manassas, VA). CHLA-259^40^ was obtained from www.COGcell.org. The human DAOY cell line used as a control with *MYC* amplified cell lines was derived from a desmoplastic medulloblastoma and harboring a C242F *p53* mutation^41^,^42^ were obtained from American Type Culture Collection (Manassas, VA) and cultured in MEM media containing 1% L-glutamine and 10% fetal bovine serum (FBS). Immortalized human benign meningothelial meningioma cells (BMEN1)^27^ were used here as a model of normal meningothelial cells that were obtained from DSMZ animal cell culture collection (Braunschweig, Germany) and cultured in DMEM containing 1% L-glu and 20% FBS. Fibroblastic human meningeal cells (HMC) were obtained from ScienCell Research Lab (cat #1400, Carlsbad, CA) and cultured in Meningeal Cell Medium (MenCM, Cat. No. 1401) supplemented with meningeal cell growth supplement (MCGS, Cat. No. 1452) and 2% FBS according to the manufacturer’s instructions. To prepare the conditioned media, 500,000 cells were seeded in a 10 cm culture dish and incubated at standard culture conditions. After 2 days, cells were washed with phosphate buffer saline (PBS) solution and added with 10 ml serum free MEM basal media and incubation was continued for 4 additional days. After 4 days of culture, the supernatant was centrifuged at 450 gs for 5 min and stored in aliquots at −80 °C. MC65 human neuroblastoma cells were cultured as previously described^43^ in MEMalpha supplemented with 10% FBS and 1 μg/ml tetracycline.

### Fluorescence *In Situ* Hybridization (FISH)

Cytogenetics were performed at the OHSU Research Cytogenetics Laboratory. See also legend for Supplemental Fig. S2.

### Chemical Screens

A panel of 60 drugs (Supplemental Table S1) was developed containing compounds of interest and/or those in the pediatric clinical development pipeline. Each compound was plated in four concentrations (10 μM, 1 μM, 100 nM, 10 nM) in triplicate in 384-well format. Medulloblastoma cell lines were plated at a density of 8000/well for D425 cells, 10,000/well for CHLA-01-MED and 25000/well for D341 cells and incubated at 37 °C with humidified 5% CO_2_ for three days. Cell viability was determined by CellTiter96® AQueous MTS Reagent (cat. G1111, Promega, Madison, WI) per manufacturer’s instructions and absorbance was measured using a BioTek Synergy 2 plate reader (BioTek, Winooski, VT USA). IC50 values were determined by a nonlinear best fit method.

### Immunocytochemistry and Immunohistochemistry

For immunocytochemistry, cytospin preparations of BMEN1 cells were analyzed by the OHSU Department of Pathology using Ventana V9 anti-Vimentin on a Vantan XT instrument according to the manufacturer standard protocol (Ventana Medical Systems, Tucson, AZ). For immunohistochemistry of meningiomas and medulloblastomas and associated meninges, 7-micron sections were prepared from formalin-fixed, paraffin-embedded tissues. Deparaffinized sections were blocked and treated with antibodies to MMP9 (EP1255Y from Novus Biologicals, Littleton, CO), Vimentin (V9 from Ventana), CD68 (KP-1 from Ventana), Collagen IV (CIV-22 from Cell Marque, Rocklin, CA), CD34 (QBEND-10 from Ventana), factor VIII (rabbit polyclonal antibody from Cell Marque), or CD61 (2F-2 from Cell Marque). MMP9 was used at 1:500 dilution and developed by hand using Vector Red alkaline phosphatase substrate (Vector Laboratories, Burlingame, CA). All other immunohistochemical stains were performed on a Ventana Benchmark XT autostainer according to protocols specifiied by the manufacturer, and developed using diaminobenzidine chromogen.

### qRT-PCR

Analyses were performed by Taqman assays (PE Applied Biosystems, Foster City, CA) for the genes of interest with normalization to *GAPDH* and relative quantification calculated by the delta (delta Ct) method. Primer and probe pairs were *S100A4* (Hs00243202_m1), *TEK* (Hs00945146_m1), *GFAP* (Hs00909233_m1) and *GAPDH* (Hs02758991_g1).

### Migration Assays

Migration assays were performed using the Polyester Membrane Transwell-Clear Inserts (cat. 3472; Corning, Tewksbury, MA). Top wells were plated at a density of 1.5 × 10^5^ 24 h serum-starved D425 cells or 1.0 × 10^5^ 24 h serum-starved CHLA-01-MED cells/well in 50 μl. IGF1 (cat. 291-G1-200; R&D Systems, Minneapolis, MN) was added at different concentrations (0 to 100 ng/ml) to bottom wells. The relative migration was determined after 48 h of incubation with IGF1 using CellTiterGlo luminescent assay (cat. G7572, Promega) per manufacturer’s instructions and measured with the IVIS Lumina II imaging system.

### Cell Adhesion Assays

D425 cells were serum deprived for 24 hours, collected by centrifugation, and re-suspended in serum free media at a density of 5,000,000 cells/mL. Calcein AM was added to a final concentration of 5 μM. Cells were incubated at 37 °C for 30 minutes, washed three times with pre-warmed serum-free media, and plated on pre-coated microplates containing the leptomeningeal extracellular matrix components laminin, fibronectin, and collagen type IV or the human cell lines BMEN or HMC cells. BMEN and HMC cells were plated at a cell density of 10,000 cells/well and allowed to establish for 24 hours prior to experimentation. D425 cells were plated at a cell density of 70,000 cells per well. To examine the effect of IGF1 on ECM and cell-cell adhesion, rhIGF1 (cat. 291-G1-200, R&D Systems, Minneapolis, MN) was added to a concentration of 10 ng/mL. Plates were incubated at 37 °C for 60 minutes. Total fluorescence was measured using a Synergy BioTek HT using the 485 nm excitation filter and the 528 nm emission filter with gain set to 55. Non-adherent cells were removed by washing microplates x 4 with PBS. Fluorescence was measured as above. Dividing post-wash fluorescence by pre-wash fluorescence and multiplying by 100 calculated percent adherent cells. Statistical comparisons were made by Student’s t-test.

### Bioactivity of IGF1 cleaved from IGF1-IGFBP3 complexes by MMP9

D425 cells were serum-deprived for 24 hours. rhMMP9 (cat. 911-MP,R&D Systems) was diluted to 100 μg/mL in 50 mM Tris, 10 mM NaCl, 0.05% Brij-35 (w/v), pH 7.5 (TCNB buffer) and activated by adding AMPA to a final concentration of 1 mM and incubated at 37 °C for 24 hours. rhIGFBP3 (cat. 675-B3,R&D Systems) and rhIGF1 (cat. 291-G1-200, R&D Systems) were preincubated for 60 minutes at 37 °C with or without activated MMP9 in cleavage buffer (150 mM NaCl, 10 mM HEPES (pH 7.4), 5 mM CaCl2 for 60 mins. Incubation concentrations for rhMMP9, rhIGF1, and rhIGFBP3 were, 20 ng/mL, 10 ng/mL, 20 ng/mL, respectively. rhIGF1, rhIGFBP3, rhMMP9, a mixture of rhIGF1 and rhIGFBP3 with and without activated rhMMP9 were added to serum-derpived D425 cells and incubated for 60 minutes. Cells were lysed in radioimmunoprecipitation buffer containing protease and phosphatase inhibitors. Phospho-IGF1R was measured by ELISA per manufacturer’s protocol (cat. 7820S, Cell Signaling, Danvers, MA) Briefly, cell lysates were diluted to 0.5 mg/mL in PathScan Sample Diluent. 100 μL of each sample was added to a microwell plate coated with Phospho-IGF-1 Receptor β (Tyr1131) rabbit antibody and incubated overnight at 4 °C. The plate was washed 4 times in PathScan 1X Wash Buffer. IGF-1 Receptor mouse antibody was added to each well and incubated at 37 °C for 1 hour. Plate was washed as before. Anti-mouse IgG, HRP-linked antibody was added to each well and incubated at 37 °C for 30 minutes and rinsed in PBS. PathScan TMB Substrate was added to each well and incubated at 37 °C for 10 minutes. PathScan® Stop Solution was added to each well. Absorbance was read at 450 nm using the Synergy BioTek HT plate reader. The significance of variation in absorbance with treatment was assessed with a one-way analysis of variance. Contrasts with the control (SFM) were corrected for multiple comparisons using Dunnet’s method and other contrasts were corrected using Tukey’s method. Absorbance was log_10_ transformed prior to analysis and all statistical testing was two-sided with an experiment-wise significance level of 5%. R was used throughout.

### DNA Sequencing

DNA was purified from tumor cell lysates using a Qiagen mini-kit. Sequencing was performed by preparing a library of targeted exons for a panel of 37 genes (Supplemental Table S2) using custom primers (Ion Torrent AmpliSeq). The resulting amplicons were modified with adapters and barcodes and then subjected to emulsion PCR before being loaded on a 318 semiconductor sequencing chip and sequenced on an Ion Torrent PGM.

### Immunoblotting

Tumors were lysed in radioimmunoprecipitation assay (RIPA) buffer containing both protease and phosphatase inhibitor (Sigma Aldrich, St. Louis, MI). The lysates were homogenized and centrifuged at 8000 g for 10 minutes. The resulting supernatants were used for immunoblot analysis with rabbit anti-phospho IGFIRβ antibody (cat. Sc-101703, Santa Cruz Biotechnology, Santa Cruz, CA), rabbit anti-IGFIRβ antibody (cat. Sc-713, Santa Cruz), mouse anti-β-actin antibody (cat. A1978, Sigma-Aldrich, St. Louis, MO). Patient-derived tumors were used for immunoblot analysis with rabbit anti-IGFIRβ antibody (cat. 3027S, cell signaling). For western blotting of MMP9 and tPA, flasks of confluent cells were washed with PBS, scraped into 10 ml PBS, collected by centrifugation at 800 g for 5 minutes, and lysed in the 62.5 mM Tris-HCl, pH 6.8, 25% glycerol, and 2% sodium dodecyl sulfate component of Laemmli sample buffer without bromphenol blue or DTT. Lysates were clarified by centrifugation at 12,000 g for 10 minutes. A bicinchoninic assay (BCA) was performed on supernatants which were normalized for protein content, colored with addition of bromphenol blue to 0.05%, reduced with addition of DTT to 50 mM, and heated to 95 degrees C for 5 minutes. Twenty-five μg of protein from each extract was electrophoresed in 7.5% polyacrylamide gels. After transfer to PVDF membranes, western blotting was performed using mouse anti-MMP9 antibody (4A3) (cat. NBP2-13173, Novus Biologicals, Littleton, CO) at 1:500 dilution and rabbit anti-tPA antibody (cat. GTX103453, Genetex, Irvine, CA) at 1:500 dilution and developed by enhanced chemiluminescence (BioRad Clarity Western ECL Substrate, cat.170–5061, Hercules, CA) according to the manufacturer’s specifications.

### mRNA Microarray Analysis

We accumulated a pool of gene expression values for cancerous samples and normal samples from published datasets^44^,^45^. The tumor set consists of 62 human medulloblastoma samples of differing subtypes^45^ (GEO accession number GSE12992)and the normal set consists of 57 normal human cerebellum samples from patients younger than 35 years of age^44^ (GEO accession number GSE15745). To perform differential expression analysis, each dataset was first normalized to the global mean across all genes and samples. The key genes were extracted from the resulting normalized datasets. The medulloblastoma samples used the Affymetrix Human Genome U133 Plus 2.0 Array, and thus had multiple probes for each gene. The normal samples used the Illumina human ref. 46 v2.0 expression beadchip, and had a single expression value for each gene. Z-scores for each gene probe in each tumor sample were calculated using 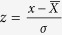, where *x* is the normalized gene probe expression, 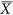 and *σ* are the mean and standard deviation of the normalized gene expression in the pooled normal samples, respectively. Two tailed student’s t-tests were used to compare the cohort gene probe expression with the cohort normal gene expression.

### Statistical Analysis

Except as stated, all data are presented as mean ± sd. One-way ANOVA was used to determine statistical significance of expression analysis and *in vitro* assay. P < 0.05 was considered statistically significant.

## Additional Information

**How to cite this article**: Svalina, M. N. *et al.* IGF1R as a Key Target in High Risk, Metastatic Medulloblastoma. *Sci. Rep.* **6**, 27012; doi: 10.1038/srep27012 (2016).

## Supplementary Material

Supplementary Information

Supplementary Information

## Figures and Tables

**Figure 1:**
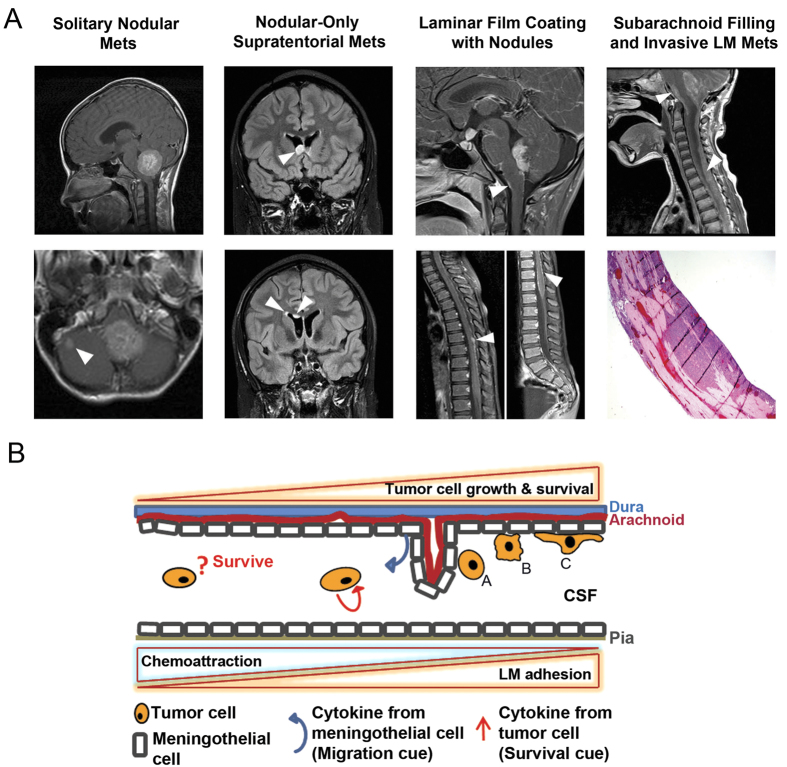
Clinical and Biological Aspects of Leptomeningeal Metastasis. (**A**) Clinical patterns of medulloblastoma metastasis. Columns from left to right, infratentorial intracerebellar solitary nodular metastasis (T1 post-gadolinium), multiple supratentorial intraventricular nodular metastases (FLAIR post-gadolinium in a patient with *P53* deleted medulloblastoma), concurrent laminar coating and nodules of the brainstem and spinal leptomeninges (T1 post-gadolinium), aggressive continuous metastases filling the subarachnoid space of the brainstem and spinal cord (T1 post-gadolinium). Mets, metastases. LM, leptomeningeal. (**B**) Schematic concept-of-hypothesis showing possible events in the process of leptomeningeal metastasis.

**Table 1:** Features of CSF Samples.

Specimen	Risk Category	Clinical Features	Age (years, months)	CSF IGF1 (ng/mL)	CSF IGFBP3 (ng/mL)
*Medulloblastoma*					
PCB-00201	Standard	desmoplastic/nodular histology	1y9m	4.35	5.8
PCB-00006	Standard	grade IV, no anaplasia	12y	9.81	5.4
PCB-00041	High	no metastasis	6y	6.22	6.2
PCB-00101	High	grade IV, moderate anaplasia	8y	3.18	9.2
PCB-00063	High	recurrent, progressive with ventricular and leptomeningeal metastasis yet indolent disease	18y	0.20	5.65
PCB-00180	High/Very High	leptomeningeal metastasis, recurrent metastasis	9y	7.22	5.95
PCB-00144	High/Very High	severe anaplasia, focal large cell features	4y	6.72	9.15
PCB-00205	High/Very High	–	4y	14.73	23.85
*Acute Lymphoblastic Leukemia*					
PCB-00163	–	active disease at CSF acquisition then remission; never CNS+	2y	1.14	5.4
PCB-00164	–	remission at CSF acquisition and thereafter; never CNS+	2y	3.78	4.8
PCB-00166	–	active disease at CSF acquisition then remission; never CNS+	7y	4.07	5.4
PCB-00167	–	active disease at CSF acquisition and thereafter; never CNS+	9y	6.47	5.6
PCB-00169	–	remission at CSF acquisition and thereafter; never CNS+	7y	1.88	5.1

**Table 2:** Features of Metastatic Medulloblastoma Cell Lines.

Cell Line	Culture Growth	Gender	Dx Age (yr)	Source	*c-MYC* amplified?	*N-MYC* Amplified?	*P53* status	Reference
D425	suspension	male	6	CNS	Yes (double minutes)	No (but 4 chromo 2. near-tetraploid)	V274C # (hemi/homozygous)	38
D341++(#)	suspension	male	3.5	CNS (yet extra-axial metastasis to abdomen, marrow)	Yes (homologous staining region, repeats on single chromo.)	No (but reportedly over-expresses N-MYC)	wildtype #	39
CHLA-01-MED	suspension	male	8	CNS (disseminated)	Yes (double minutes)	No	wildtype #	n/a (ATCC)
CHLA-259*	suspension & adherent	unknown	14	CNS (no LM mets, anaplastic histology)	Unknown (very little expression)	Unknown (over-expresses N-MYC)	Wildtype^40^	40

Dx, diagnosis. Yr, years. Chromo, chromosome. LM, leptomeningeal. Met, metastasis. ^*^Not used in this study, but included for reference. ^#^as determined by IonTorrent sequencing assay^53^. A full listing of sequencing panel results is given in Supplemental Table S2. ++This cell line harbors TSC2 A415V (heterozygous; unknown variant; not reported in COSMIC).

## References

[b1] Partap S. *et al.* Medulloblastoma incidence has not changed over time: a CBTRUS study. Journal of pediatric hematology/oncology 31, 970–971 (2009). 19887963 10.1097/MPH.0b013e3181bbc502

[b2] Fruhwald M. C. & Plass C. Metastatic medulloblastoma--therapeutic success through molecular target identification? Pharmacogenomics J 2, 7–10 (2002). 11990385 10.1038/sj.tpj.6500077

[b3] Packer R. J., Cogen P., Vezina G. & Rorke L. B. Medulloblastoma: clinical and biologic aspects. Neuro Oncol 1, 232–250 (1999). 11550316 10.1215/15228517-1-3-232 PMC1920747

[b4] Partap S. *et al.* Medulloblastoma incidence has not changed over time: a CBTRUS study. J Pediatr Hematol Oncol 31, 970–971 (2009). 19887963 10.1097/MPH.0b013e3181bbc502

[b5] Roussel M. F. & Hatten M. E. Cerebellum development and medulloblastoma. Curr Top Dev Biol 94, 235–282 (2011). 21295689 10.1016/B978-0-12-380916-2.00008-5 PMC3213765

[b6] Geyer J. R. *et al.* Multiagent chemotherapy and deferred radiotherapy in infants with malignant brain tumors: a report from the Children’s Cancer Group. Journal of clinical oncology: official journal of the American Society of Clinical Oncology 23, 7621–7631 (2005). 16234523 10.1200/JCO.2005.09.095

[b7] Korah M. P. *et al.* Incidence, risks, and sequelae of posterior fossa syndrome in pediatric medulloblastoma. Int J Radiat Oncol Biol Phys 77, 106–112 (2010). 19695790 10.1016/j.ijrobp.2009.04.058

[b8] Kortmann R. D. *et al.* Postoperative neoadjuvant chemotherapy before radiotherapy as compared to immediate radiotherapy followed by maintenance chemotherapy in the treatment of medulloblastoma in childhood: results of the German prospective randomized trial HIT ‘91. Int J Radiat Oncol Biol Phys 46, 269–279 (2000). 10661332 10.1016/s0360-3016(99)00369-7

[b9] Dufour C. *et al.* Metastatic Medulloblastoma in Childhood: Chang’s Classification Revisited. Int J Surg Oncol 2012, 245385 (2012). 22312539 10.1155/2012/245385 PMC3265270

[b10] Taylor M. D. *et al.* Molecular subgroups of medulloblastoma: the current consensus. Acta Neuropathol 123, 465–472 (2012). 22134537 10.1007/s00401-011-0922-z PMC3306779

[b11] Del Valle L. *et al.* Insulin-like growth factor I receptor activity in human medulloblastomas. Clinical cancer research : an official journal of the American Association for Cancer Research 8, 1822–1830 (2002). 12060623

[b12] Northcott P. A. *et al.* Medulloblastomics: the end of the beginning. Nature reviews. Cancer 12, 818–834 (2012). 23175120 10.1038/nrc3410 PMC3889646

[b13] Lastowska M. *et al.* Identification of a neuronal transcription factor network involved in medulloblastoma development. Acta Neuropathol Commun 1, 35 (2013). 24252690 10.1186/2051-5960-1-35 PMC3893591

[b14] de Pablo F. & de la Rosa E. J. The developing CNS: a scenario for the action of proinsulin, insulin and insulin-like growth factors. Trends Neurosci 18, 143–150 (1995). 7754526 10.1016/0166-2236(95)93892-2

[b15] Browd S. R. *et al.* N-myc can substitute for insulin-like growth factor signaling in a mouse model of sonic hedgehog-induced medulloblastoma. Cancer Research 66, 2666–2672 (2006). 16510586 10.1158/0008-5472.CAN-05-2198

[b16] Kawauchi D. *et al.* A mouse model of the most aggressive subgroup of human medulloblastoma. Cancer Cell 21, 168–180 (2012). 22340591 10.1016/j.ccr.2011.12.023 PMC3285412

[b17] Northcott P. A. *et al.* Medulloblastoma comprises four distinct molecular variants. J Clin Oncol 29, 1408–1414 (2011). 20823417 10.1200/JCO.2009.27.4324 PMC4874239

[b18] Weller R. O. Microscopic morphology and histology of the human meninges. Morphologie 89, 22–34 (2005). 15943078 10.1016/s1286-0115(05)83235-7

[b19] Beschet I., Brunon J., Scoazec J. Y. & Mosnier J. F. Expression of beta1 and beta4 integrins in normal arachnoid membrane and meningiomas. Cancer 86, 2649–2658 (1999). 10594860

[b20] Baia G. S. *et al.* A genetic strategy to overcome the senescence of primary meningioma cell cultures. J Neurooncol 78, 113–121 (2006). 16554968 10.1007/s11060-005-9076-y

[b21] Kartenbeck J., Schwechheimer K., Moll R. & Franke W. W. Attachment of vimentin filaments to desmosomal plaques in human meningiomal cells and arachnoidal tissue. J Cell Biol 98, 1072–1081 (1984). 6365927 10.1083/jcb.98.3.1072 PMC2113124

[b22] Hardy S. J., Christodoulides M., Weller R. O. & Heckels J. E. Interactions of Neisseria meningitidis with cells of the human meninges. Mol Microbiol 36, 817–829 (2000). 10844670 10.1046/j.1365-2958.2000.01923.x

[b23] Culig Z. Cytokine disbalance in common human cancers. Biochim Biophys Acta 1813, 308–314 (2011). 21167870 10.1016/j.bbamcr.2010.12.010

[b24] Sciume G., Santoni A. & Bernardini G. Chemokines and glioma: invasion and more. J Neuroimmunol 224, 8–12 (2010). 20656128 10.1016/j.jneuroim.2010.05.019

[b25] Bunn R. C., King W. D., Winkler M. K. & Fowlkes J. L. Early developmental changes in IGF-I, IGF-II, IGF binding protein-1, and IGF binding protein-3 concentration in the cerebrospinal fluid of children. Pediatric research 58, 89–93 (2005). 15774848 10.1203/01.PDR.0000156369.62787.96

[b26] Christodoulides M. *et al.* Interaction of Neisseria meningitidis with human meningeal cells induces the secretion of a distinct group of chemotactic, proinflammatory, and growth-factor cytokines. Infect Immun 70, 4035–4044 (2002). 12117909 10.1128/IAI.70.8.4035-4044.2002 PMC128145

[b27] Puttmann S. *et al.* Establishment of a benign meningioma cell line by hTERT-mediated immortalization. Lab Invest 85, 1163–1171 (2005). 15965488 10.1038/labinvest.3700307

[b28] Takenaga K. *et al.* Modified expression of Mts1/S100A4 protein in C6 glioma cells or surrounding astrocytes affects migration of tumor cells *in vitro* and *in vivo*. Neurobiol Dis 25, 455–463 (2007). 17223348 10.1016/j.nbd.2006.10.021

[b29] Das A., Tan W. L. & Smith D. R. Expression of extracellular matrix markers in benign meningiomas. Neuropathology 23, 275–281 (2003). 14719542 10.1046/j.1440-1789.2003.00512.x

[b30] Kim J. M. *et al.* Curcumin suppresses the TPA-induced invasion through inhibition of PKCalpha-dependent MMP-expression in MCF-7 human breast cancer cells. Phytomedicine 19 (2012). 10.1016/j.phymed.2012.07.002 22921746

[b31] Martin D. C., Fowlkes J. L., Babic B. & Khokha R. Insulin-like growth factor II signaling in neoplastic proliferation is blocked by transgenic expression of the metalloproteinase inhibitor TIMP-1. J Cell Biol 146, 881–892 (1999). 10459021 10.1083/jcb.146.4.881 PMC2156132

[b32] Wu X. *et al.* Clonal selection drives genetic divergence of metastatic medulloblastoma. Nature 482, 529–533 (2012). 22343890 10.1038/nature10825 PMC3288636

[b33] Boccaccio C. *et al.* The MET oncogene drives a genetic programme linking cancer to haemostasis. Nature 434, 396–400 (2005). 15772665 10.1038/nature03357

[b34] Elzi D. J. *et al.* Plasminogen activator inhibitor 1–insulin-like growth factor binding protein 3 cascade regulates stress-induced senescence. Proc Natl Acad Sci USA 109, 12052–12057 (2012). 22778398 10.1073/pnas.1120437109 PMC3409757

[b35] Ohshima-Hosoyama S., Hosoyama T., Nelon L. D. & Keller C. IGF-1 receptor inhibition by picropodophyllin in medulloblastoma. Biochemical and biophysical research communications 399, 727–732 (2010). 20692232 10.1016/j.bbrc.2010.08.009

[b36] Del Valle L. *et al.* Insulin-like growth factor I receptor activity in human medulloblastomas. Clin Cancer Res 8, 1822–1830 (2002). 12060623

[b37] Ramaswamy V. *et al.* Recurrence patterns across medulloblastoma subgroups: an integrated clinical and molecular analysis. Lancet Oncol 14, 1200–1207 (2013). 24140199 10.1016/S1470-2045(13)70449-2 PMC3953419

[b38] He X. M. *et al.* Differentiation characteristics of newly established medulloblastoma cell lines (D384 Med, D425 Med, and D458 Med) and their transplantable xenografts. Lab Invest 64, 833–843 (1991). 1904513

[b39] Friedman H. S. *et al.* Phenotypic and genotypic analysis of a human medulloblastoma cell line and transplantable xenograft (D341 Med) demonstrating amplification of c-myc. Am J Pathol 130, 472–484 (1988). 3279793 PMC1880676

[b40] Xu J. *et al.* Novel cell lines established from pediatric brain tumors. J Neurooncol 107, 269–280 (2012). 22120608 10.1007/s11060-011-0756-5 PMC3379550

[b41] Jacobsen P. F., Jenkyn D. J. & Papadimitriou J. M. Establishment of a human medulloblastoma cell line and its heterotransplantation into nude mice. J Neuropathol Exp Neurol 44, 472–485 (1985). 2993532 10.1097/00005072-198509000-00003

[b42] Raffel C., Thomas G. A., Tishler D. M., Lassoff S. & Allen J. C. Absence of p53 mutations in childhood central nervous system primitive neuroectodermal tumors. Neurosurgery 33, 301–305, discussion 305–306 (1993). 8396224 10.1227/00006123-199308000-00018

[b43] Woltjer R. L. *et al.* Role of glutathione in intracellular amyloid-alpha precursor protein/carboxy-terminal fragment aggregation and associated cytotoxicity. J Neurochem 93, 1047–1056 (2005). 15857408 10.1111/j.1471-4159.2005.03109.x

[b44] Gibbs J. R. *et al.* Abundant quantitative trait loci exist for DNA methylation and gene expression in human brain. PLoS Genet 6, e1000952 (2010). 20485568 10.1371/journal.pgen.1000952 PMC2869317

[b45] Kool M. *et al.* Molecular subgroups of medulloblastoma: an international meta-analysis of transcriptome, genetic aberrations, and clinical data of WNT, SHH, Group 3, and Group 4 medulloblastomas. Acta Neuropathologica 123, 473–484 (2012). 22358457 10.1007/s00401-012-0958-8 PMC3306778

[b46] Gajjar A. *et al.* Risk-adapted craniospinal radiotherapy followed by high-dose chemotherapy and stem-cell rescue in children with newly diagnosed medulloblastoma (St Jude Medulloblastoma-96): long-term results from a prospective, multicentre trial. Lancet Oncol 7, 813–820 (2006). 17012043 10.1016/S1470-2045(06)70867-1

[b47] Ara T. *et al.* Immunohistochemical expression of MMP-2, MMP-9, and TIMP-2 in neuroblastoma: association with tumor progression and clinical outcome. Journal of pediatric surgery 33, 1272–1278 (1998). 9722003 10.1016/s0022-3468(98)90167-1

[b48] Goussev S. *et al.* Differential temporal expression of matrix metalloproteinases after spinal cord injury: relationship to revascularization and wound healing. J Neurosurg 99, 188–197 (2003). 12956462 10.3171/spi.2003.99.2.0188 PMC2792200

[b49] Noble L. J., Donovan F., Igarashi T., Goussev S. & Werb Z. Matrix metalloproteinases limit functional recovery after spinal cord injury by modulation of early vascular events. J Neurosci 22, 7526–7535 (2002). 12196576 10.1523/JNEUROSCI.22-17-07526.2002 PMC2792199

[b50] Sulik A. & Chyczewski L. Immunohistochemical analysis of MMP-9, MMP-2 and TIMP-1, TIMP-2 expression in the central nervous system following infection with viral and bacterial meningitis. Folia histochemica et cytobiologica / Polish Academy of Sciences, Polish Histochemical and Cytochemical Society 46, 437–442 (2008). 10.2478/v10042-008-0058-8 19141395

[b51] Levin E. G., Banka C. L. & Parry G. C. Progressive and transient expression of tissue plasminogen activator during fetal development. Arterioscler Thromb Vasc Biol 20, 1668–1674 (2000). 10845887 10.1161/01.atv.20.6.1668

[b52] Ware J. H., Dibenedetto A. J. & Pittman R. N. Localization of tissue plasminogen activator mRNA in adult rat brain. Brain Res Bull 37, 275–281 (1995). 7627570 10.1016/0361-9230(95)00008-3

[b53] Beadling C. *et al.* Combining highly multiplexed PCR with semiconductor-based sequencing for rapid cancer genotyping. The Journal of molecular diagnostics: JMD 15, 171–176 (2013). 23274167 10.1016/j.jmoldx.2012.09.003

