# Supplementary material for: IGF1R as a Key Target in High Risk, Metastatic Medulloblastoma
Source: Sci Rep. 2016 Jun 3;6:27012. doi: 10.1038/srep27012 (PMC4891740; doi:10.1038/srep27012)
Supplement: Supplementary Information [file srep27012-s1.pdf]

IGF1R as a Key Target in High Risk, Metastatic Medulloblastoma

Running Title: IGF1R modulates MYC+ medulloblastoma migration and growth

Matthew N. Svalina[1,2], Ken Kikuchi[1], Jinu Abraham[1], Sangeet Lal[1], Monika A. Davare[1], Teagan P. Settelmeyer[2], Michael C. Young[2], Jennifer L. Peckham[1], Yoon-Jae Cho[1,3], Joel E. Michalek[4], Brian S. Hernandez[4], Noah E. Berlow[1], Melanie Jackson[1], Daniel J. Guillaume[5], Nathan R. Selden[5], Darell D. Bigner[6], Kellie J. Nazemi[7], Sarah C. Green[8], Christopher L. Corless[8], Sakir Gultekin[8], Atiya Mansoor[8], Brian P. Rubin[9], Randall Woltjer[8], Charles Keller[1,2*]

[1] Department of Pediatrics, Oregon Health & Science University, Portland, OR 97239 USA

[2] Children's Cancer Therapy Development Institute, Beaverton, OR USA

[3] Division of Child Neurology, Stanford Medicine Cancer Institute, Palo Alto, CA 94304 USA

[4] Department of Epidemiology and Biostatistics, University of Texas Health Science Center, San Antonio, TX 78229 USA

[5] Division of Pediatric Neurosurgery, Department of Neurological Surgery, Oregon Health & Science University, Portland, OR 97239 USA

[6] Pediatric Brain Tumor Foundation Institute at Duke, Duke University Medical Center, Durham, NC 27705 USA

[7] Department of Pediatrics, Oregon Health & Science University Doernbecher Children's Hospital, Portland, OR 97239 USA

[8] Department of Pathology, Oregon Health & Science University, Portland, OR 97239 USA.

[9] Departments of Anatomic Pathology and Molecular Genetics, Taussig Cancer Center and Lerner Research Institute, Cleveland Clinic Foundation, Cleveland, OH 44195 USA

* corresponding author: Charles Keller, Children's Cancer Therapy Development Institute, 12655 S.w. Beaverdam Rd W, Beaverton, OR 97005 USA, Tel (970) 239-4296, Fax (970) 237-6388, email charles@cc-tdi.org

[abstract 149 words; text 5498 words excluding title page, abstract, references and figure legends; 7 Figures; 2 Tables; 2 Supplemental Figures; 2 Supplemental Tables]

SUPPLEMENTAL MATERIAL

Supplemental Figure Legends

Supplemental Figure 1. (A) Summary of all results for human angiogenesis profile arrays for CSF samples. (B) Summary of all results for human cytokine profile arrays for CSF samples. (C) Summary of all results for human angiogenesis profile arrays for medulloblastoma cell lines, a benign meningioma cell line, and a human meningeal cell line. (D) Summary of all results for human cytokine arrays for medulloblastoma cell lines, a benign meningioma cell line, and a human meningeal cell line.

Supplemental Figure S2. MYCC and MYCN status of medulloblastoma cell lines. (left colum) representative appearance in culture. (middle column) *C-MYC* FISH using Abbott probes for c-MYC (LSI C-MYC, orange) with an identifier probe for the chromosome 8 centromeric region (8 alpha satellite, aqua). (right column) *N-MYC* FISH using Abbott probes for N-MYC (LSI N-MYC, green) with an identifier probe for the chromosome 2 centromeric region (CEP2, orange).

Supplemental Figure S3. (A) Heatmap of gene expression for tumor vs. normal z-scores of selected genes in mutliple published medulloblastoma samples[51,52]. These data were generated via gene expression microarrays and thus multiple probes are associated with each gene. **(B, C)** Histology of the meninges for PCB-205 showed no microscopic tumor involvement, but evidence of inflammatory processes as demonstrated by the presence of focal hemosiderin (brown pigment) and focal histiocytes and lymphocytes.

Supplemental Tables

Supplemental Table S1. Customized Drug Screen

Drug	Target	Supplier	Catalog Number	Validation
5-aza-cytidine	RRM2	Sigma-Aldrich	A2385-100MG	HPLC, NMR
ABT-888 (veliparib)	PARP inhibitor	Selleck	S1004	HPLC/NMR
actinomycin-D	DNA damage by binding transcription initiation complex	Sigma	A9415	HPLC, NMR
AP24534 (ponatinib)	pan-BCR-ABL inhibitor; also inhibits VEGFR2, FGFR1, PDGFRa, mutant FLT3, and LYN	Selleck	S1491	HPLC/NMR
AZD1152-HQPA (barasertib)	selective inhibitor of Aurora B, may also inhibit STK6	Selleck	S1147	HPLC, NMR
AZD6244 (selumetinib)	MEK-1/2 inhibitor	Selleck	S1008	HPLC/NMR
BIX 02188	MEK5 selective inhibitor	Selleck	S1530	HPLC/NMR
BIX01294	histone-lysine methyltransferase inhibitor	Tocris	3364	TLC, HPLC, NMR, Mass Spectrum
bleomycin sulfate	DNA damage	Selleck	S1214	HPLC/NMR
BMS 754807	inhibits IGF1R and IR	Jihe Pharma	JH-1013	HPLC, NMR
bortezomib	proteasome inhibitor	Selleck	S1013	HPLC/NMR
bosutinib (SKI-606)	Src/Abl inhibitor	Selleck	S1014	HPLC/NMR
carboplatin	DNA damage	Santa Cruz Biotech	SC-202093	
carfilzomib	epoxyketone-based irreversible proteasome inhibitor	Chemietek	CT-CARF	HPLC
cediranib (AZD2171)	VEGFR inhibitor	Selleck	S1017	HPLC/NMR
chromeceptin	IGF attenuation by binding MFP-2, stimulating IGFBP-1 and SOCS-3 to activate STAT6	Sigma	C0868	HPLC, NMR, Mass Spectrum
cisplatin	uses platinum to cross-link DNA	Selleck	S1166	HPLC, NMR
crizotinib	ALK inhibitor	Selleck	S1068	HPLC/NMR
curcumin	interferes with NF-kB activity, may inhibit mTOR complex I, multiple cell proliferation pathway kinases	Selleck	S1848	HPLC, NMR

dasatinib	BCR/ABL and Src inhibitor	Selleck	S1021	HPLC/NMR
EKB-569	EGFR tyrosine kinase inhibitor	Selleck	S1198	HPLC/NMR
enzastaurin (LY317615)	PKC-b inhibitor	Selleck	S1055	HPLC/NMR
erlotinib hydrochloride	EGFR inhibitor	Selleck	S1023	HPLC/NMR
GANT61	inhibits hedgehog by blocking GLI function	Tocris	3191	HPLC, NMR, Mass Spectrum
GDC-0449 (vismodegib)	inhibits hedgehog receptors PTCH and/or SMO, ABCG2, Pgp, MRP1	Selleck	S1082	HPLC/NMR
imatinib (STI571)	BCR/ABL inhibitor, maybe PDGF-Rb also	Selleck	S2475	HPLC/NMR
INCB018424 (ruxolitinib)	JAK 1 and 2 inhibitor	Selleck	S1378	HPLC, NMR
irinotecan	topoisomerase 1 inhibitor	Selleck	S1198	HPLC/NMR
itraconazole	inhibits hedgehog	Selleck	S2476	HPLC/NMR
lapatinib ditosylate	EGFR and HER2/neu inhibitor	Selleck	S1028	HPLC/NMR
LBH-589 (panobinostat)	histone deacetylase inhibitor	Selleck	S1030	HPLC/NMR
methazolastone (Temozolomide)	DNA alkylation	Tocris	2706	HPLC, NMR, Mass Spectrum
methotrexate	competitive inhibitor of DHFR	Selleck	S1210	HPLC/NMR
MK-2206	Akt inhibitor	Selleck	S1078	HPLC/NMR
MLN8237	inhibits aurora A, upregulates p53, p21, p27	Selleck	S1133	HPLC/NMR
N-acetyl cysteine	free radical scavenger	Sigma-Aldrich	A7250	Infrafed spectrum, TLC
nicotinamide (niacinamide)	inhibits PARP-1 enzymes	Selleck	S1899	HPLC/NMR
obatoclax mesylate (GX15-070)	pan-Bcl-2 inhibitor	Selleck	S1057	HPLC/NMR
OSI-906	inhibits IGF1R and IR	Chemietek	CT-O906	HPLC
pazopanib Hydrochloride	inhibits VEGFR-1, VEGFR-2, VEGFR-3, PDGFR-a/b, and c-kit	Selleck	S1035	HPLC/NMR
PD0332991	inhibits CDK4 and CDK6	Selleck	S1116	HPLC/NMR
PI-103	PI3K inhibitor	Selleck	1038	HPLC/NMR
picropodophyllin	inhibits IGF1R	Southwest Research Institute	09-0203-007-002	NMR
prednisone (adasone)	inhibits transcription of COX-2, cytokines, cell adhesion molecules, inducible NO synthetase; blocks D3-mediated induction of osteocalcin in Ob, modifies collegenase	Selleck	S1622	HPLC/NMR

	gene transcription; increases annexin-1			
quinacrine dihydrochloride	non-selective MAO-A/B inhibitor	Sigma	Q3251	Infrared spectrum, TLC area
rapamycin (sirolimus)	inhibits cytokine production in response to IL-2 and cell motility (mTOR-mediated S6K1 and 4E-BP1 pathways)	Selleck	S1139	HPLC/NMR
resveratrol	upregulation of p21, p53, and Bax; down-regulation of survivin, cyclin D1, cyclin E, Bcl-2, Bcl-xL, and cIAPs; activation of capsases; suppression of NF-kB, AP-1, Egr-1; inhibits JNK, PAPK, Akt, PKC, PKD, and casein kinase II; down-regulation of COX-2, 5-LOX, VEGF, IL-1, IL-6, IL-8, AR, PSA	Selleck	S1396	HPLC/NMR
RO4929097	gamma secretase inhibitor that blocks activation of Notch receptors	Selleck	S1575	HPLC/NMR
SJ-172550	inhibits MDMX	Tocris	T7329	HPLC, NMR, Mass Spectrum
SNS-032 (BMS-387032)	inhibits CDK 2, 7, and 9; upregulates p57	Selleck	S1145	HPLC/NMR
sodium valproate (valproic acid)	histone deacetylase inhibitor	Selleck	S1168	HPLC/NMR
sodium aurothiomalate	decreases inflammation with unknown mechanism	Sigma	157201-1G	Infrared spectrum
sodium butyrate	histone deacetylase inhibitor	Selleck	S1999	HPLC/NMR
sorafenib tosylate (Bay 43-9006)	inhibits Raf-1, BRAF, BRAF(V599E), VEGFR-2, VEGFR-3, PDGFR-b, Flt-3, and c-kit	Selleck	S1040	HPLC/NMR
SP600125	inhibits JNK-1, 2, and 3; minor inhibition of other kinases	Selleck	S1460	HPLC/NMR
sunitinib malate (sutent)	inhibits PDGF-Rs, VEGFRs, KIT(CD117), RET, CSF-1R, Flt3	Selleck	S1542	HPLC/NMR
taurolidine	PARP cleavage in some cells but not others	Sigma	T7329	NMR
temsirolimus	specific inhibitor of mTOR	Selleck	S1044	HPLC/NMR
trichostatin A	histone deacetylase inhibitor	Selleck	S1046	HPLC/NMR

vincristine Sulfate	binds to tubulin dimers, inhibiting microtubule assembly	Selleck	S1241	HPLC/NMR
vorinostat(SAHA)	histone deacetylase inhibitor	Selleck	S1047	HPLC/NMR
VX-680 (tozasertib)	inhibits aurora A, B, C; Flt-3; and Abl	Selleck	S1048	HPLC/NMR
zibotentan (ZD4054)	ETA-receptor antagonist	Selleck	S1456	HPLC/NMR

Supplemental Figure S1

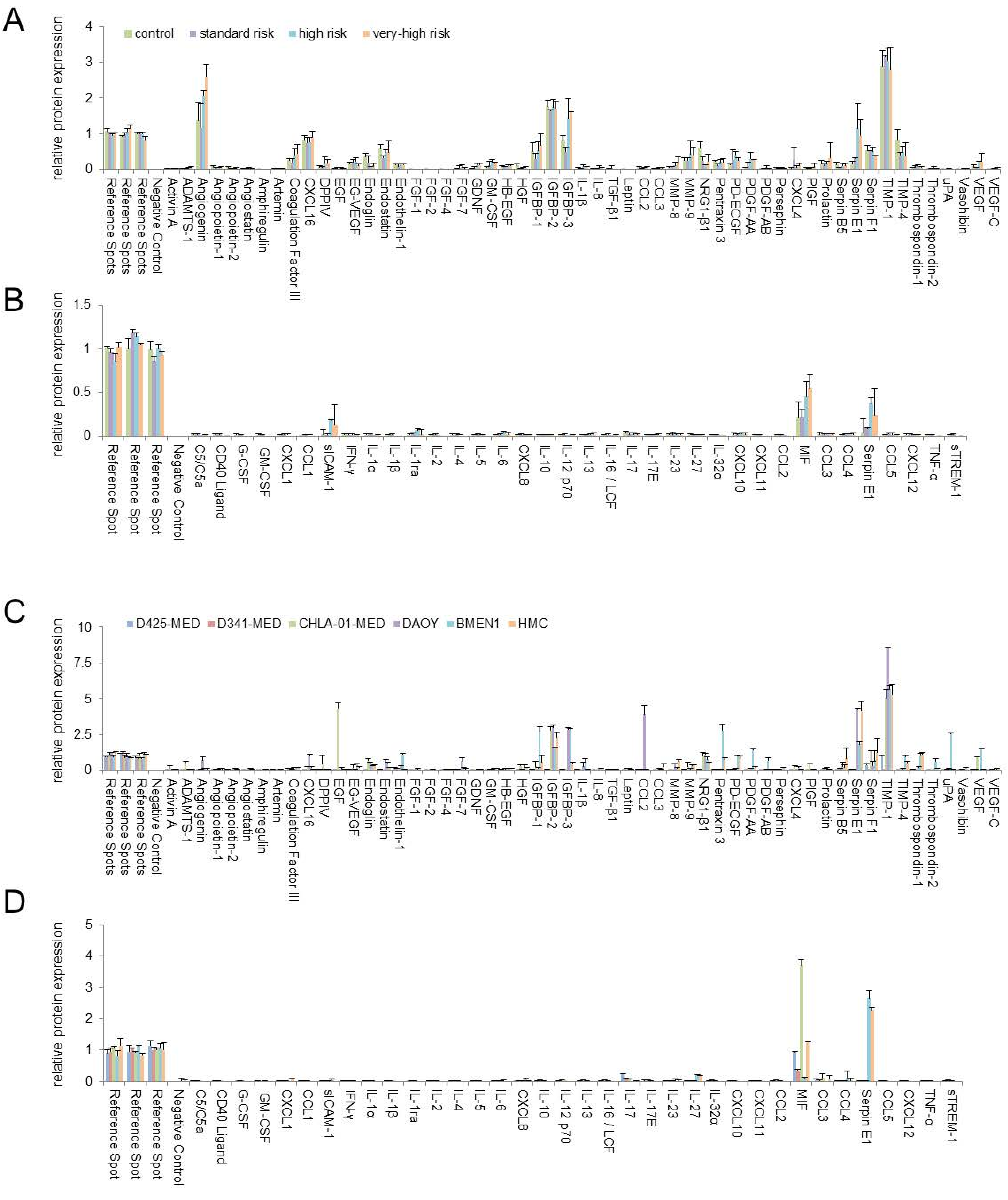

Supplemental Fig S2

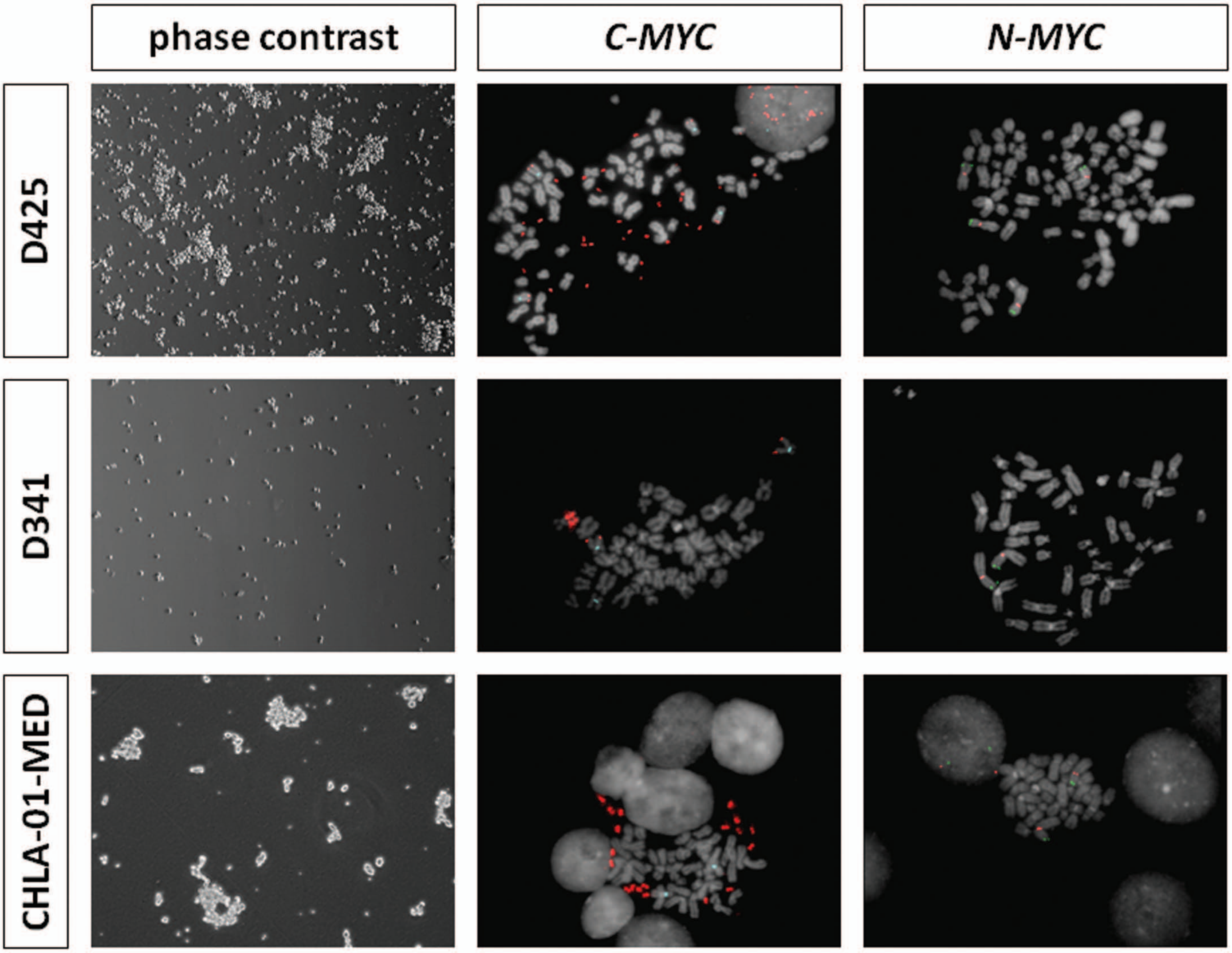

Supplemental Figure S3

A

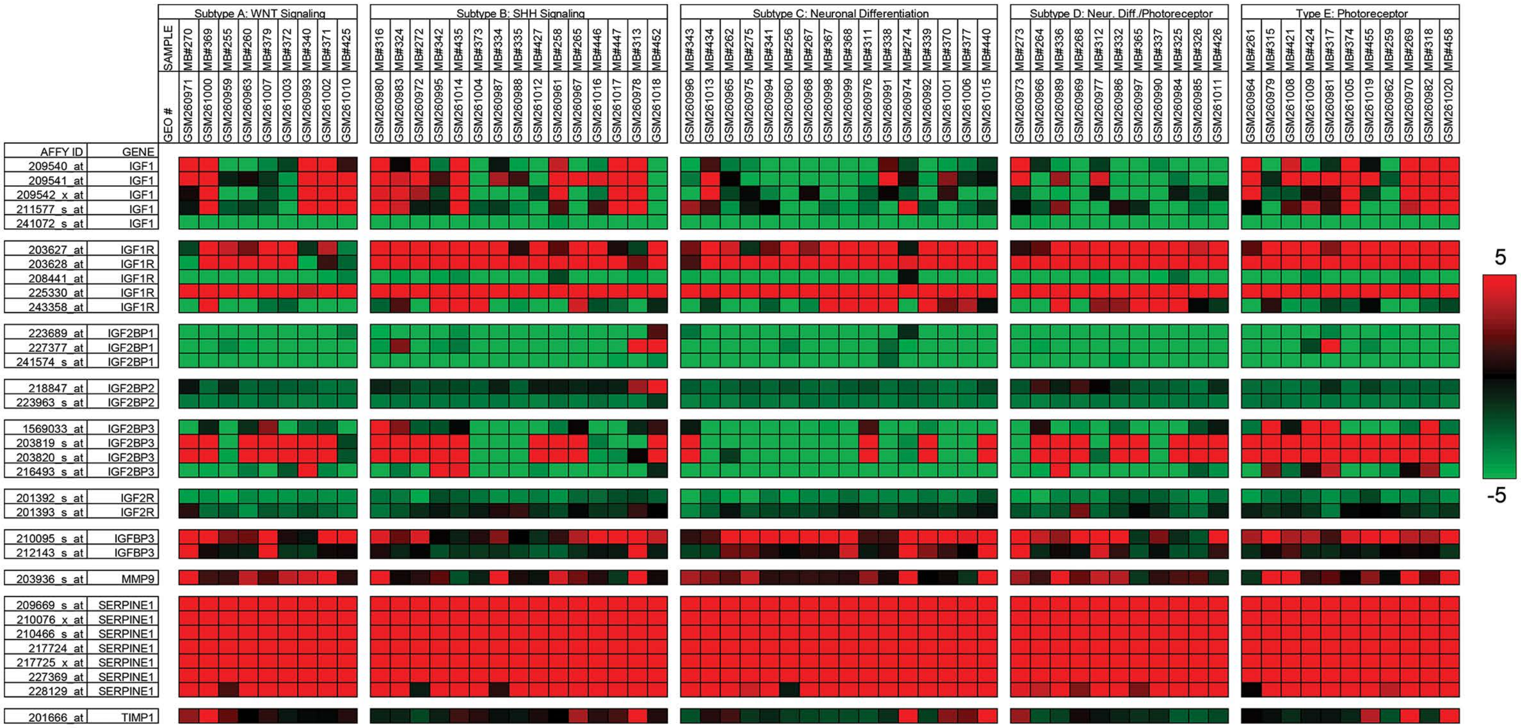

B

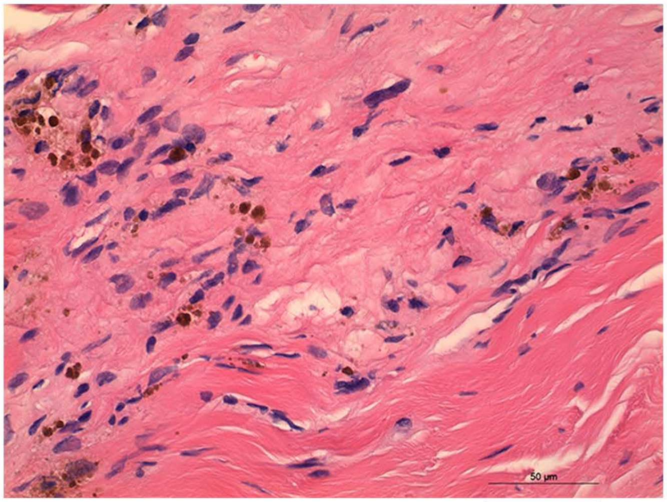

50 µM

C

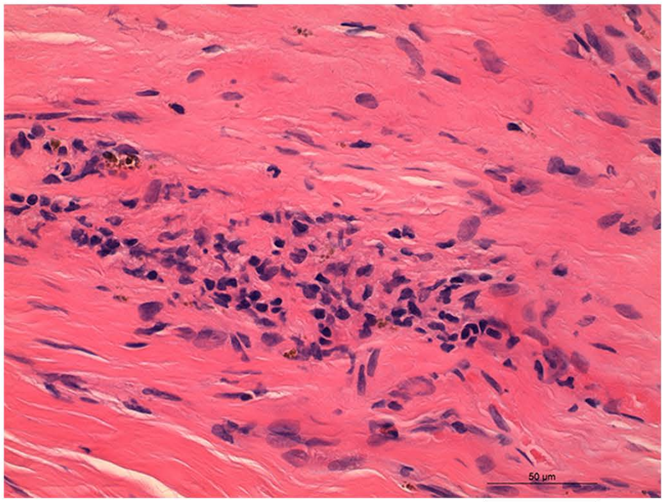

50 µM
